# Effect of Fermented Rapeseed Meal in Diets for Piglets on Blood Biochemical Parameters and the Microbial Composition of the Feed and Faeces

**DOI:** 10.3390/ani12212972

**Published:** 2022-10-28

**Authors:** Łukasz Wlazło, Bożena Nowakowicz-Dębek, Mateusz Ossowski, Marcin Łukaszewicz, Anna Czech

**Affiliations:** 1 Department of Animal Hygiene and Environmental Hazards, Faculty of Animal Sciences and Bioeconomy, University of Life Sciences in Lublin, 13 Akademicka, 20-950 Lublin, Poland; 2 Department of Biotransformation, Faculty of Biotechnology, University of Wroclaw, F. Joliot-Curie 14A, 50-383 Wroclaw, Poland; 3 Department of Biochemistry and Toxicology, Faculty of Animal Sciences and Bioeconomy, University of Life Sciences in Lublin, 13 Akademicka, 20-950 Lublin, Poland

**Keywords:** piglets, fermented rapeseed meal, faeces, feed, microbes, biochemical parameters

## Abstract

**Simple Summary:**

To relieve stress during the weaning of piglets, pig farmers use functional feed additives that improve gastrointestinal function. Fermented products show this kind of potential. Fermented rapeseed meal is a source of valuable protein and also has a positive effect on animals’ health parameters and gastrointestinal microbiota. In this work, we demonstrated that fermentation is an effective way to improve the nutritional value of rapeseed meal. The results also showed that the presence of lactic acid bacteria in diets containing a fermented component had a positive effect on metabolic processes in pigs. As the content of lactic acid is stable over time, it favourably influences the feed microbiota, which translates into animal health.

**Abstract:**

The study assessed the influence of rapeseed meal (RSM) fermented using *Bacillus subtilis* 87Y on the feed microbiota, intestinal microbiota, blood biochemical parameters, and content of minerals in the blood plasma and faeces of piglets. Modulation of the microbial composition of feed containing fermented rapeseed meal (FRSM) and of the faeces of pigs consuming it was observed. There was a significant increase in the number of lactic acid bacteria (LAB) and a decrease in the total number of coliforms and *Clostridium perfringens* in the faeces of animals from the experimental groups. FRSM in the diet of piglets was shown to improve the mineral balance by increasing the levels of P, Ca, and Mg in the blood plasma and reducing their amount in the faeces. A beneficial effect on parameters of protein and lipid metabolism was also noted, resulting in an increase in the levels of total protein (TP) and albumins (ALB) and a reduction in triacylglycerols (TG) and low-density lipoprotein (LDL) cholesterol in the blood plasma of the piglets. The research results indicate that the presence of FRSM in the diet of weaners can be a preventive factor in intestinal dysbiosis and support the maintenance of homeostasis.

## 1. Introduction

Rapeseed is a plant with very high feed potential due to its high content of digestible, bioavailable and palatable nutrients. The use of rapeseed meal (RSM) in the diet of various groups of animals is limited by the presence of substances that reduce or prevent the utilisation of nutrients or have a harmful effect on the animal, such as glucosinolates, tannins, or phytic acid [1]. An effective means of reducing undesirable substances in RSM, including phytic acid, glucosinolates, and dietary fibre, is fermentation [2]. The fermentation process is usually carried out using bacteria of the genus *Lactobacillus* or fungi *Aspergillus niger* [3,4,5]. Other strains that could improve the efficiency of the fermentation process are sought as well [6]. One such strain is *Bacillus subtilis* 87Y. *Bacillus subtilis* is generally recognised as safe (GRAS) and is known as a producer of extracellular enzymes. As a non-pathogenic probiotic, *Bacillus subtilis* can be used as a feed additive to improve intestinal function in animals. It is believed to improve nutrient digestion and absorption as well as the bacterial balance in the intestines [7,8]. It directly stimulates animals’ growth and improves their health [9,10,11]. Antimicrobial peptides produced by *Bacillus subtilis*, owing to their high level of activity and bactericidal capacity, are promising therapeutic tools. This is particularly important in light of the growing problem of antibiotic resistance among bacteria and the ban on their use, as *Bacillus subtilis* antimicrobial peptides can play an increasing role in treating bacterial infections.

*Bacillus subtilis* 87Y has also been shown to significantly improve the characteristics of the fermented product. Our previous research demonstrated that it caused an increase in the protein content of RSM and an over six-fold increase in the level of lactic acid while reducing the content of dietary fibre and crude fat [12]. Thus, the use of this strain for the production of a fermented feed component based on RSM can be an alternative to soybean meal (SBM) and can help to fight pathogens in animals. Literature reports indicate that fermented rapeseed meal (FRSM), as a source of pro-prebiotic substances, microbial enzymes [13], short-chain organic acids [14] and antioxidants [15], can be an alternative to currently used feed additives in compound feeds.

To date, no studies have been conducted on the use of rapeseed fermented with *Bacillus subtilis* strain 87Y (FRSMb) in the diet of piglets. Analysis of the literature indicates that the use of fermented components in the diet of pigs has a beneficial effect by reducing the frequency of diarrhoea in young animals and the frequency of swine dysentery [13,16,17], as well as improving homeostasis of the body. According to many authors, this is the result of the proliferation of populations of microbes inseparably associated with the fermented material [3]. This microbiota consists mainly of lactic acid bacteria (LAB) and yeast, but may also include coliform bacteria, *Salmonella* and moulds. During fermentation, LAB convert sugars mainly to lactic acid and acetic acid. This causes a decrease in the substrate pH, and the environment becomes unfavourable to contaminants such as *Enterobacteriaceae* [3].

Due to the interesting composition of FRSMb, we have conducted a series of experiments using it as a component of feed for mink [12], rabbits [18,19], and quail [20]. The inclusion of FRSMb in the diet of mink and rabbits improved the quality and hygiene of the feed as well as the physiology (including biochemical parameters of the plasma), microbiology and morphology of the digestive tract. In addition, the reduction in antinutrients present in RSM (phytic acid, glucosinolate, and non-starch polysaccharides) and the presence of microbial enzymes produced during fermentation increased the utilisation of nutrients contained in the feed, especially minerals [21]. This is particularly important in young animals, whose need for bioavailable nutrients and minerals is very high and whose gastrointestinal tract (GIT) is highly sensitive to the presence of antinutrients [13].

As the results of our previous research on mink and rabbits were very promising, we decided to test the effectiveness of FRSMb in diets for piglets during their most sensitive period, i.e., after weaning. We hypothesised that the immunomodulatory properties of fermented components [21] would make it possible to omit zinc from the diet of weaners without adversely affecting metabolic processes in the body, as suggested by Satessa et al. [4] and Satessa et al. [22]. Analysis of these parameters may be an important contribution to the formulation of diets for piglets. Therefore, the aim of the study was to analyse the effect of RSM fermented using *Bacillus subtilis* 87Y on the microbial composition of the feed, modulation of the intestinal microbiota, biochemical parameters of the blood, and the content of minerals in the animals’ plasma and faeces.

## 2. Materials and Methods

All animal protocols in this study were approved by the Local Ethics Committee on Animal Experimentation of the University of Life Sciences in Lublin, Poland (approval no. 50/2018, of 1 April 2018), Poland.

### 2.1. Preparation of Fermented Rapeseed Meal (FRSM)

Fermented rapeseed meal was obtained by means of fermentation using *Bacillus subtilis* strain 87Y from the strain collection of InventionBio sp. z o.o. (Bydgoszcz), [23].

### 2.2. Experimental Design

A total of 288 crossbred (Yorkshire × Danish × Landrace) growing pigs [144 barrows and 144 gilts; average body weight (BW) 7 kg] were randomly assigned to three dietary treatments (six replicates with eight barrows and eight gilts per replicate). The experiment was begun when the pigs were 28 days old (after weaning) and continued until they reached a BW of about 35 kg (when they were transferred to the fattening room).

### 2.3. Animal Diets

The dietary treatments were a control diet (C) based on maize, wheat and SBM, which contained all nutrients in levels recommended by the NRC [24]. The diet for group FRA was based on the control diet but with 8% FRSMb in place of some of the SBM. Group FR also received a diet with 8% FRSMb in place of some of the SBM, but without enzyme additives, pro- and prebiotic additives, organic acids, or added zinc. The experiment was carried out in farm conditions, and for hygiene and economic reasons the owners of the farm did not consent to the use of negative control, i.e., a diet without zinc oxide or any additives improving the animal’s immune function. The ingredient composition and nutrient content of the diets are presented in Table 1 [23]. Piglets were provided ad libitum access to water during the entire experimental trial.

The additive was homogenised with the bulk feed mass and then subjected to a technological agglomeration process to produce feed pellets to be dispensed through the feed line.

### 2.4. Analysed Nutrient Levels in Experimental Diets

Diets for chemical and microbiological analysis were sampled twice, at the beginning and end of the experiment. Some of the results for nutrients and minerals contained in the diets are presented by Czech et al. [23].

Percentage differences in the contents of nutrients, antinutrients and bioactive substances with respect to the control are presented in Table 2.

### 2.5. Sampling and Measurements

#### 2.5.1. Diets and Faeces

At 20–22 kg and 30–35 kg BW, faeces were sampled from 6 weaners from each group for mineral analysis. To this end, the weaners were placed in metabolic cages (a separate cage for each individual). For four days faeces were collected at the same time of day and then weighed and oven-dried to determine the dry weight (DW). These samples were used for the analysis of mineral contents.

At the same time, each diet was sampled twice for chemical analysis. To determine the content of minerals (calcium, manganese, magnesium, chromium, sodium, and potassium), the samples were mineralised and analysed using the PlasmaQuant PQ 9000 (Analytik Jena, Jena, Germany) inductively coupled plasma optical emission spectrometer. Total phosphorus content was determined according to Fiske and Subbarow [25].

HPLC–MS/MS was used to analyse the content of mycotoxins in the diets: aflatoxin B1, B2, G1, and G2, ochratoxin A, deoxynivalenol, and zearalenone. Samples for toxicological analysis were crushed in an Ultra Centrifugal Mill ZM 200 (Retsch, Haan, Germany) and then subjected to extraction and purification using Bond Elut^®^ Mycotoxin columns (Varian, Darmstadt, Germany) and internal standards (IS). The purified extracts were analysed using a liquid chromatograph—Agilent 1200 Series Gradient HPLC System (Agilent Technologies, Santa Clara, CA, USA)—with a mass spectrometry detector—3200 QTRAP^®^ LC-MS/MS System (Sciex, Framingham, MA, USA). For a more thorough assessment of feed quality, antioxidant parameters of the diets were determined: total phenolic content according to Szydłowska-Czerniak and Tułodziecka [26], amount of 2,2-Diphenyl-1-picrylhydrazyl (DPPH) radical according to Abeysekera et al. [27], and ferric-reducing antioxidant power (FRAP) according to Szydłowska-Czerniak et al. [28]. All analyses were performed in triplicate.

At the same time, at BW of 20–22 kg and 30–35 kg, samples of faeces from the pigs in metabolic cages and samples of feed (about 50 g) were collected for microbiological analysis. Previously prepared feed was sampled from six bags, with randomisation of the sample. Faeces were sampled directly from the rectum of six weaners from each group. The material was placed in sterile containers and immediately chilled to 6–8 °C. After being transported to the laboratory, each sample was homogenised using a Unidrive X1000 Homogeniser Drive (CAT Scientific, Staufen, Germany). Then, 20 g of homogenised material was weighed out and placed in 180 mL of Ringer solution with Tween 80. The stock solution was shaken for 15 min and left to stand for another 5 min. A series of decimal dilutions of the solution was prepared according to ISO 6887 [29], after which each sample was plated on previously prepared sterile solid media in an inoculation volume of 100 μL and then incubated according to standards ISO 4833 [30], ISO 21527 [31], ISO 4832 [32], ISO 16649 [33], and ISO 7937 [34]. The results were calculated as colony-forming units per g (cfu/g) of material.

#### 2.5.2. Blood Plasma

At the end of the experiment (about 35 kg BW), blood samples (10 mL) were collected from one randomly selected barrow from each replicate pen for plasma biochemical parameters (3 groups × 6 replicates). The animals had no access to feed for 12 h before blood was drawn. Samples were collected by anterior vena cava puncture using a 22-gauge sterile needle. Blood samples were collected into tubes containing heparin. The plasma was then separated by centrifugation for 10 min at 3500 rpm and 4 °C, and then stored at −80 °C until biochemical analysis.

#### 2.5.3. Blood Plasma and Faecal Chemical Analysis

The concentrations of glucose (GLU), TP, albumins (ALB), total cholesterol (TC), high-density lipoprotein cholesterol (HDL), triacylglycerols (TG) and phosphorus in the blood plasma were determined by spectrophotometry using tests by Cormay. The content of low-density lipoprotein cholesterol (LDL) was calculated. The activity of alanine aminotransferase (ALT), aspartate aminotransferase (AST), and alkaline phosphatase (ALP) were determined by spectrophotometry using tests by Cormay.

All spectrophotometric analyses were performed using the Unicam 5625 UV/VIS Spectrophotometer (Unicam Ltd., Cambridge, United Kingdom). To determine the content of minerals (calcium, manganese, magnesium, chromium, sodium, and potassium), the samples were mineralised and analysed using the PlasmaQuant PQ 9000 (Analytik Jena, Jena, Germany) inductively coupled plasma optical emission spectrometer.

### 2.6. Statistical Analysis

The results obtained in each group are presented as means, *p*-values and standard error of the mean (SEM). The significance of differences between groups was determined by the post hoc Tukey test, for significance levels of 0.05 and 0.01. The analysis was performed using Statistica software ver. 10.0 (StatSoft S.A., Tulsa, OK, USA). Means within a row marked with different letters (a, b) differ significantly at *p* ≤ 0.05.

## 3. Results

The total phenolic content in the diets with FRSMb (groups FRA and FR) was more than 70% higher than in the control diet. The highest FRAP value and DPPH level were noted in group FRA, and the lowest in the control (Table 2). The level of mycotoxins did not exceed acceptable limits in any of the diets used in the experiment (Table 2).

The FR diet had a significantly lower number of fungi than the diets for groups FRA and C. The total number of fungi was significantly lower in the diet for group FRA compared with group C. The diet for piglets from groups FRA and FR had a significantly higher total number of LAB compared with the control (*p* < 0.001). It is worth noting that the group C diet contained no LAB (Table 3). These relationships were observed at the beginning and end of the experiment.

At the start of the experiment, the total number of mesophilic bacteria and the total number of LAB of the genus *Lactobacillus* was significantly higher in the faeces of piglets from groups FRA and FR than in the faeces of the control group (*p* = 0.034 and *p* = 0.033, respectively). After two weeks of the experiment, the total number of mesophilic bacteria was the highest and the total number of fungi was the lowest in the faeces of piglets from group FR compared with FRA and C (*p* = 0.003 and *p* = 0.045, respectively). The total number of coliforms and the total number of *Clostridium perfringens* in the faeces was highest in group C compared with the other groups (*p* ≤ 0.05). The faeces of piglets from groups FRA and FR had a significantly higher total number of *Lactobacillus* bacteria than in the control group (*p* = 0.009), (Table 4).

The content of TP and albumins was significantly higher in the plasma of piglets from groups FRA and FR than in the controls. Total cholesterol and HDL content were significantly higher in group FR than in group C (*p* < 0.001 and *p* = 0.032, respectively). The plasma LDL and TG levels and CHOL/HDL ratio were significantly lower in piglets from groups FRA and FR compared with controls (*p* = 0.003 and *p* = 0.048, respectively), while the HDL level was significantly higher (*p* < 0.001). Alanine aminotransferase activity levels in the plasma of piglets from groups FRA and FR were significantly lower compared with piglets from group C. Alkaline phosphatase activity in group FR was significantly higher than in the other experimental groups, while AST activity was significantly the highest in group FRA (Table 5).

The plasma of piglets from groups FRA and FR had a significantly higher content of phosphorus, calcium, and magnesium compared with the control group (*p* < 0.015). Only the content of chromium was significantly higher in the plasma of piglets from the control group compared with group FRA (*p* = 0.042), (Table 6).

The faeces of the piglets from group FRA contained significantly lower levels of minerals, i.e., P, Ca, Mg, Mn, and Cr, than in the control group (*p* < 0.001). A significantly lower level of Cr was also noted in the faeces of piglets from group FR compared with group C (*p* < 0.001), (Table 6).

## 4. Discussion

Fermentation is an effective means of improving the nutritional value of RSM. The effectiveness of fermentation depends on the microorganisms used. Some microbes only improve physicochemical properties, while others positively influence biological properties, and others can improve both [35]. Research has demonstrated these effects of fermentation [36]. 

According to research by Grela et al. [21], fermented products stimulate immunity in animals through probiotic and prebiotic effects. By breaking down antinutritional substances, they allow the nutritional potential of rapeseed meal to be fully utilised. Maintenance of intestinal homeostasis translates into health status parameters, as illustrated by favourable blood biochemical parameters. In the present study, there was also a marked improvement in the physicochemical and biological properties of feed containing RSM fermented using *Bacillus subtilis* 87Y, including an increase in the microbiological activity of phytase (group FRA), a reduction in the level of phytin phosphorus (groups FRA and FR), and increased antioxidant activity, mainly due to increased levels of polyphenols, tannins, and bioactive compounds present in the fermented component (groups FRA and FR). It was also demonstrated that the presence of LAB in diets with a fermented component, owing to the reduced pH, is conducive to the safe storage of feed, and thus improves its hygienic properties. Both at the start of the experiment and after 14 days of storage, the number of beneficial *Lactobacillus* bacteria was significantly higher in the diets with FRSMb than in the control diet. While no pathogenic organisms were detected in any of the diets, it is worth noting that the content of fungi was significantly higher in the diets with FRSMb. According to van Winsen et al. [37], lactic acid and other short-chain fatty acids generated during fermentation reduce the levels of *Enterobacteriaceae* and *Salmonella* spp. The undissociated form of lactic acid is believed to be responsible for reducing *Enterobacteriaceae* because it can freely pass through the bacterial membrane.

Bunte et al. [38] showed that the presence of LAB in the feed also helps to create an optimal environment for the development of beneficial microorganisms in the host GIT, which undoubtedly improves its functioning and minimises the development of pathogens. This was confirmed in our experiment. The presence of LAB in the diets with FRSMb corresponded with a significant reduction in the number of *Clostridium perfringens* in the faeces. A decline in pH to < 4.5 prevents the development of undesirable microorganisms in the intestines, such as enteropathogenic strains of *E. coli*, *Salmonella* and *Klebsiella* [39]. Enteropathogenic *E. coli* can cause neonatal and post-weaning diarrhoea (PWD) in pigs, but also has zoonotic potential. *Lactobacillus* bacteria colonising the intestinal mucosa compete with opportunistic microbes in the gut, thereby enhancing local immunity to infection [37]. Another mechanism explaining the reduction in pathogenic bacteria is the increased digestibility of nutrients in the small intestine due to fermentation of the feed, which reduces the number of substrates for the growth of microbes in the lower GIT [40]. However, the effect of lactobacilli associated with feed on the bacterial microbiota of the gut is not fully understood.

The presence of organic acids such as lactic acid in feed reduces the pH of the digesta and is conducive to phytase activity, resulting in improved availability and more efficient absorption of minerals [41]. This is confirmed by the results of our experiment. The increased availability of minerals such as phosphorus, calcium and magnesium is illustrated by their higher content in the plasma of weaners receiving a diet with FRSMb. It should be emphasised that the content of minerals in the plasma was within reference ranges [42]. The improvement in mineral absorption corresponded with a reduction in their excretion into the environment in the faeces. *Lactobacillus* bacteria, owing to their ability to convert simple sugars to lactic acid, acidify the intestinal environment, creating an optimal environment for the breakdown of phytin complexes, which directly improves the availability of minerals and reduces their levels in the faeces [41,43,44]. Similar observations following the use of FRSM were made in earlier studies [45]. The bioavailability of minerals for the host may also be determined by metabolic processes in the intestinal microbiome. This process most likely takes place gradually, as the animal acquires the ability to assimilate nutrients contained in the diet. Each section of the intestine is colonised by a variety of microbes, whose development depends on anatomical and physiological factors and on physicochemical processes [13,46]. An example is the biotransformation of exogenous polyphenols of plant origin, which owing to their antioxidant and anti-inflammatory properties stimulate the development of the intestinal microbiota, thereby improving mineral absorption by the host [47]. It should be noted that the diets with FRSMb had significantly higher concentrations of antioxidant compounds, as evidenced by the much higher content of polyphenols and parameters indicative of antioxidant capacity, i.e., DPPH and FRAP.

The presence of LAB in the feed is also conducive to pepsin activity and improves protein utilisation [48,49]. The inclusion of FRSMb in the study caused an increase in TP and ALB in the blood plasma of the piglets, which may confirm that the protein is better assimilated from the feed. Better digestibility of protein from diets with a fermented component was demonstrated in a study by Czech et al. [17]. Diets with fermented components providing easily digestible protein can modify the composition of the intestinal microbiota [50] and the secretion of pancreatic enzymes, which is linked to the quantity and qualitative composition of ingested proteins [51]. The activity of enzymes indicative of normal liver function in the plasma of the weaners receiving a diet with FRSMb did not deviate from reference values [42]. However, the significantly higher ALP activity in the weaners from group FR may have been due to increased ingestion of minerals taking part in the ossification process [52], resulting from the introduction of fermented products rich in enzymes releasing minerals from the feed (phytase and enzymes hydrolysing non-starch polysaccharide fractions).

Fermented components are also thought to play a role in regulating lipid metabolism. According to Hu et al. [53], FRSM reduces plasma levels of TG (an indicator of lipid metabolism in animals). Similar results were obtained in the present study. The reduction in the plasma levels of TG and LDL in the piglets receiving diets with FRSMb may have been linked to the reduction in the pH of the digesta, but was probably primarily due to the increase in the number of microbes synthesising numerous enzymes, including lipase, which is responsible for the digestibility of lipid components. Microbial activity may also have reduced faecal enzyme activity and lipid binding [54]. In addition, probiotic microbes in fermented products, by producing their own metabolites, inhibit esterification of cholesterol (CHOL) in the intestinal mucosa, thus reducing the level of its ‘bad’ fractions in the body through deconjugation and precipitation of bile acids. These functions are primarily performed by *Lactobacillus acidophilus* and *Bifidobacterium* [55]. Stimulation of metabolic processes of lipid compounds, including synthesis of low-molecular-weight HDL, was also demonstrated in a study by Hu et al. [53] in broilers, in which TC content was lower in the serum of birds fed a diet with FRSM. The authors suggest that fermented feed may have a similar effect to that of probiotics in reducing CHOL [56].

## 5. Conclusions

Fermented rapeseed meal used at a level of 8% in diets for piglets is a source of lactic acid that remains stable over time, as well as bioactive compounds which beneficially affect metabolic processes in the body. The use of FRSM in diets for piglets, owing to beneficial modulation of the composition of the microbiome, improved the metabolism of mineral elements, thus reducing their levels in the faeces. It also improved protein and lipid metabolism. It seems likely that FRSM in the diet of fatteners will be a preventive factor helping to maintain homeostasis in the body, resulting in improved growth performance as well as higher quality animal products.

## Figures and Tables

**Table 1:** Ingredient composition (% of air-dry matter) and nutrient content of piglet diets.

Ingredient	Group		
	C	FRA	FR
Wheat	60.48	68.33	58.8
Barley	20	20	20
Soybean meal, 46.5% protein	9.24	3.46	3.32
Fermented rapeseed meal (FRSMb)	0	8	8
Fish meal 65%	4	4	4
Soybean oil	2.1	2.13	2.13
Chalk	0.95	0.87	0.87
L-Lysine∙HCl, 78%	0.82	0.91	0.91
L-Threonine	0.37	0.4	0.4
DL-Methionine	0.26	0.25	0.25
NaCl	0.43	0.39	0.39
Calcium monophosphate	0.52	0.43	0.43
Premix 0.5%	0.5	0.5	0.5
Feed additives	0.33	0.33	0

Groups: C—control; FRA—weaners receiving a diet with 8% FRSMb and feed additives; FR—weaners receiving a diet with 8% fermented rapeseed meal (FRSMb) without feed additives. Premix—mineral–vitamin premix, content in 1 kg: Ca 240 g, K 1 g, Fe 20 g, Mn 11 g, Cu 2.5 g, Se 60.0 mg, I 120 mg, Co 150 mg, vit. A 1,600,000 IU, vit. D_3_ 200,000 IU, vit. E 10.0 g, vit. K 600 mg, vit. B_1_ 500 mg, vit. B_2_ 1400 mg, vit. B_6_ 800 mg, vit. B_12_ 10.0 mg, nicotinic acid 4.0 g, pantothenic acid 4 g, chloric choline 40 g, folic acid 300 mg. Content of feed additives in 1 kg feed: ZnO (78%) 0.160 g Zn; mixture of formic and propionic acid 3:1 (2.5 g); *E. coli* phytase (0.15 g—5000 FTU/g); xylanase, beta-glucanase, (0.15 g—12,200 U/g; 1520 U/g, respectively); pentosanase, hemicellulase and enzymes hydrolysing pectic substances (0.10 g); *Saccharomyces cerevisiae* (0.20 g).

**Table 2:** Nutrient content in 1 kg of fermented rapeseed meal (FRSMb) and experimental diets for piglets (%).

Nutrient	Group		
	C	FRA *	FR *
Dry matter		−0.6	0.0
Crude ash		9.7	−1.1
Crude protein		−0.4	−0.6
Crude fat		3.7	7.4
Crude fibre		9.2	11.1
ME		0.2	0.1
Total phosphorus		1.3	−0.7
Calcium		−1.0	1.0
Iron		2.5	0.5
Copper		6.7	0.0
Zinc		1.2	−76.0
Phytin phosphorus		−15.8	−17.4
Glucosinolates		877.2	1028.7
Lactic acid		496.6	465.0
Tannins		54.7	55.6
pH		−1.6	−1.3
Phytase		−0.7	−53.9
Antioxidant parameters			
Total polyphenols, g sinapic acid kg^−1^	3.43	5.89	5.61
DPPH, mmol Trolox kg^−1^	111.9	254.9	177.4
FRAP, mmol Trolox kg^−1^	67.9	182.0	101.23
Mycotoxins, µg/kg			
Aflatoxin B1	<LOQ = 1.0	<LOQ = 1.0	<LOQ = 1.0
Aflatoxin B2	<LOQ = 1.0	<LOQ = 1.0	<LOQ = 1.0
Aflatoxin G1	<LOQ = 1.0	<LOQ = 1.0	<LOQ = 1.0
Aflatoxin G2	<LOQ = 1.0	<LOQ = 1.0	<LOQ = 1.0
Ochratoxin A	<LOQ = 1.0	<LOQ = 1.0	<LOQ = 1.0
Deoxynivalenol	<LOQ = 25.0	<LOQ = 25.0	<LOQ = 25.0
Zearalenone	<LOQ = 25.0	<LOQ = 25.0	<LOQ = 25.0

ME—metabolic energy; DPPH—2,2-Diphenyl-1-picrylhydrazyl; FRAP—ferric-reducing antioxidant power. Groups: C—control; FRA—group receiving a diet based on the control diet, with 8% fermented rapeseed meal (FRSMb) in place of some of the soybean meal (SBM); FR—group receiving a diet based on the control diet, with 8% FRSMb in place of some of the SBM, but without enzyme additives, pro- and prebiotic additives, organic acids, or added zinc. * % difference in content of nutrients, minerals, and bioactive substances in experimental groups (FRA and FR) relative to control (C).

**Table 3:** Microbial composition of diets (cfu/g).

Parameter	Group			SEM	*p*-Value
	C	FRA	FR		
First sampling					
Total number of mesophilic bacteria	3.5 × 10^3^	2.5 × 10^3^	3.6 × 10^3^	252.5	0.426
Total number of fungi	3.9 × 10 ^a^	2.5 × 10 ^b^	1.0 × 10 ^c^	3.99	<0.001
Total number of coliforms	2.9 × 10^2^	3.0 × 10^2^	2.5 × 10^2^	3.99	0.436
Total number of *Escherichia coli*	ng	ng	ng	–	–
Total number of LAB of the genus *Lactobacillus*	ng ^b^	5.1 × 10^3 a^	5.4 × 10^3 a^	727.9	<0.001
Total number of *Clostridium perfringens*	ng	ng	ng	–	–
Second sampling					
Total number of mesophilic bacteria	3.3 × 10^3^	2.8 × 10^3^	3.4 × 10^3^	332.1	0.750
Total number of fungi	3.8 × 10 ^a^	2.9 × 10 ^b^	1.8 × 10 ^c^	2.66	<0.001
Total number of coliforms	2.6 × 10^2^	2.9 × 10^2^	2.6 × 10^2^	34.50	0.926
Total number of *Escherichia coli*	ng	ng	ng	–	–
Total number of LAB of the genus *Lactobacillus*	ng ^c^	4.0 × 10^3 b^	5.2 × 10^3 a^	683.4	<0.001
Total number of *Clostridium perfringens*	ng	ng	ng	–	–

LAB—lactic acid bacteria; SEM—standard error of the mean; ng—no growth; ^a–c^—values within a row with different superscripts differ significantly (*p* ≤ 0.05). Groups: C—control; FRA—group receiving a diet based on the control diet, with 8% fermented rapeseed meal (FRSMb) in place of some of the soybean meal (SBM); FR—group receiving a diet based on the control diet, with 8% FRSMb in place of some of the SBM, but without enzyme additives, pro- and prebiotic additives, organic acids, or added zinc.

**Table 4:** Microbial composition of the faeces of weaners (cfu/g).

Parameter	Group			SEM	*p*-Value
	C	FRA	FR		
First sampling					
Total number of mesophilic bacteria	8.4 × 10^4 a^	3.1 × 10^5 b^	3.3 × 10^5 b^	46,786.4	0.034
Total number of fungi	9.1 × 10^3^	1.4 × 10^4^	9.9 × 10^3^	1012.3	0.367
Total number of coliforms	1.4 × 10^5^	1.2 × 10^5^	1.2 × 10^5^	6732.8	0.117
Total number of *Escherichia coli*	1.3 × 10^5^	1.1 × 10^5^	1.1 × 10^5^	6419.4	0.617
Total number of LAB of the genus *Lactobacillus*	3.3 × 10^4 a^	5.1 × 10^5 b^	5.1 × 10^5 b^	89,247.4	0.033
Total number of *Clostridium perfringens*	3.1 × 10^5^	2.6 × 10^5^	1.6 × 10^5^	30,158.11	0.238
Second sampling					
Total number of mesophilic bacteria	6.2 × 10^5 a^	2.4 × 10^5 a^	2.2 × 10^6 b^	290,071.0	0.003
Total number of fungi	3.3 × 10^3 b^	5.6 × 10^3 c^	1.8 × 10^3 a^	580.3	0.045
Total number of coliforms	3.3 × 10^5 b^	2.1 × 10^5 a^	2.5 × 10^5 ab^	20,789.3	0.035
Total number of *Escherichia coli*	1.1 × 10^5^	1.9 × 10^5^	3.3 × 10^5^	105,633.0	0.110
Total number of LAB of the genus *Lactobacillus*	7.2 × 10^6 a^	3.3 × 10^7 b^	3.7 × 10^7 b^	4,113,519	0.009
Total number of *Clostridium perfringens*	2.1 × 10^5 c^	9.1 × 10^4 b^	2.9 × 10^4 a^	24,666.09	0.040

LAB—lactic acid bacteria; SEM—standard error of the mean. ^a–c^—values within a row with different superscripts differ significantly (*p* ≤ 0.05). Groups: C—control; FRA—group receiving a diet based on the control diet, with 8% fermented rapeseed meal (FRSMb) in place of some of the soybean meal (SBM); FR—group receiving a diet based on the control diet, with 8% FRSMb in place of some of the SBM, but without enzyme additives, pro- and prebiotic additives, organic acids, or added zinc.

**Table 5:** Biochemical parameters of the plasma of piglets.

Parameter	Group			SEM	*p*-Value
	C	FRA	FR		
GLU; mmol L^−1^	3.41	3.26	3.38	0.051	0.461
TP; g L^−1^	54.95 ^b^	62.43 ^a^	59.75 ^a^	0.944	0.049
ALB; g L^−1^	30.43 ^b^	36.88 ^a^	35.45 ^a^	0.324	0.023
CHOL; mmol L^−1^	1.90 ^b^	1.95 ^b^	2.24 ^a^	0.042	<0.001
HDL; mmol L^−1^	1.10 ^b^	1.36 ^ab^	1.61 ^a^	0.053	0.032
LDL; mmol L^−1^	0.575 ^a^	0.387 ^b^	0.424 ^b^	0.027	0.003
% HDL	58.02 ^b^	69.84 ^a^	72.05 ^a^	1.64	<0.001
CHOL/HDL	1.73 ^a^	1.43 ^b^	1.39 ^b^	0.040	<0.001
TG; mmol L^−1^	0.496 ^a^	0.446 ^b^	0.444 ^b^	0.009	0.048
ALP; U L^−1^	157.2 ^b^	178.7 ^b^	250.2 ^a^	10.68	<0.001
ALT; U L^−1^	47.97 ^a^	40.18 ^b^	38.90 ^b^	1.38	0.006
AST; U L^−1^	46.40 ^b^	57.02 ^a^	45.37 ^b^	1.54	<0.001

SEM—standard error of the mean. ^a,b^—values within a row with different superscripts differ significantly (*p* ≤ 0.05). GLU—glucose; TP—total protein; ALB—albumins; CHOL—cholesterol; HDL—high-density lipoprotein cholesterol; LDL—low-density lipoprotein cholesterol; TG—triacylglycerols; ALP—alkaline phosphatase; ALT—alanine aminotransferase; AST—aspartate aminotransferase. Groups: C—control; FRA— group receiving a diet based on the control diet, with 8% fermented rapeseed meal (FRSMb) in place of some of the soybean meal (SBM); FR—group receiving a diet based on the control diet, with 8% FRSMb in place of some of the SBM, but without enzyme additives, pro- and prebiotic additives, organic acids, or added zinc.

**Table 6:** Content of minerals in the plasma and faeces of piglets.

Parameter	Group			SEM	*p*-Value
	C	FRA	FR		
Blood plasma					
Phosphorus; mmol L^−1^	3.28 ^b^	3.48 ^a^	3.50 ^a^	0.033	0.015
Calcium; mmol L^−1^	2.99 ^b^	3.21 ^a^	3.23 ^a^	0.046	<0.001
Magnesium; mmol L^−1^	0.946 ^b^	1.00 ^a^	1.01 ^a^	0.010	0.015
Manganese; µmol L^−1^	0.218	0.243	0.230	0.008	0.519
Chromium; µmol L^−1^	0.554 ^a^	0.404 ^b^	0.495 ^ab^	0.024	0.042
Sodium; mmol L^−1^	80.02	78.21	77.53	0.205	0.061
Potassium; mmol L^−1^	4.72	4.47	4.48	0.054	0.120
Faeces (per kg DW)					
Total phosphorus, g	3.63 ^a^	2.77 ^b^	3.26 ^a^	0.088	<0.001
Calcium, g	5.55 ^a^	4.83 ^b^	5.05 ^ab^	0.079	<0.001
Magnesium, g	1.93 ^a^	1.79 ^b^	1.80 ^ab^	0.017	<0.001
Manganese, g	0.073 ^a^	0.065 ^b^	0.070 ^a^	0.001	<0.001
Chromium, mg	0.274 ^a^	0.160 ^c^	0.235 ^b^	0.012	<0.001
Sodium, g	0.240	0.255	0.267	0.003	0.058
Potassium, g	2.85	2.71	2.70	0.016	0.067

SEM—standard error of the mean. ^a–c^—values within a row with different superscripts differ significantly (*p* ≤ 0.05). DW—dry weight. Groups: C—control; FRA—group receiving a diet based on the control diet, with 8% fermented rapeseed meal (FRSMb) in place of some of the soybean meal (SBM); FR—group receiving a diet based on the control diet, with 8% FRSMb in place of some of the SBM, but without enzyme additives, pro- and prebiotic additives, organic acids, or added zinc.

## Data Availability

All relevant data are within the paper.
